# Supplementary material for: Multilocus Data Analysis Reveal the Diversity of Cryptic Species in the Tillandsia ionantha (Bromeliaceae: Tillansiodeae) Complex
Source: Plants (Basel). 2022 Jun 28;11(13):1706. doi: 10.3390/plants11131706 (PMC9269404; doi:10.3390/plants11131706)
Supplement: Supplementary file 1 [file plants-11-01706-s001.zip › plants-1754943-supplementary.pdf]

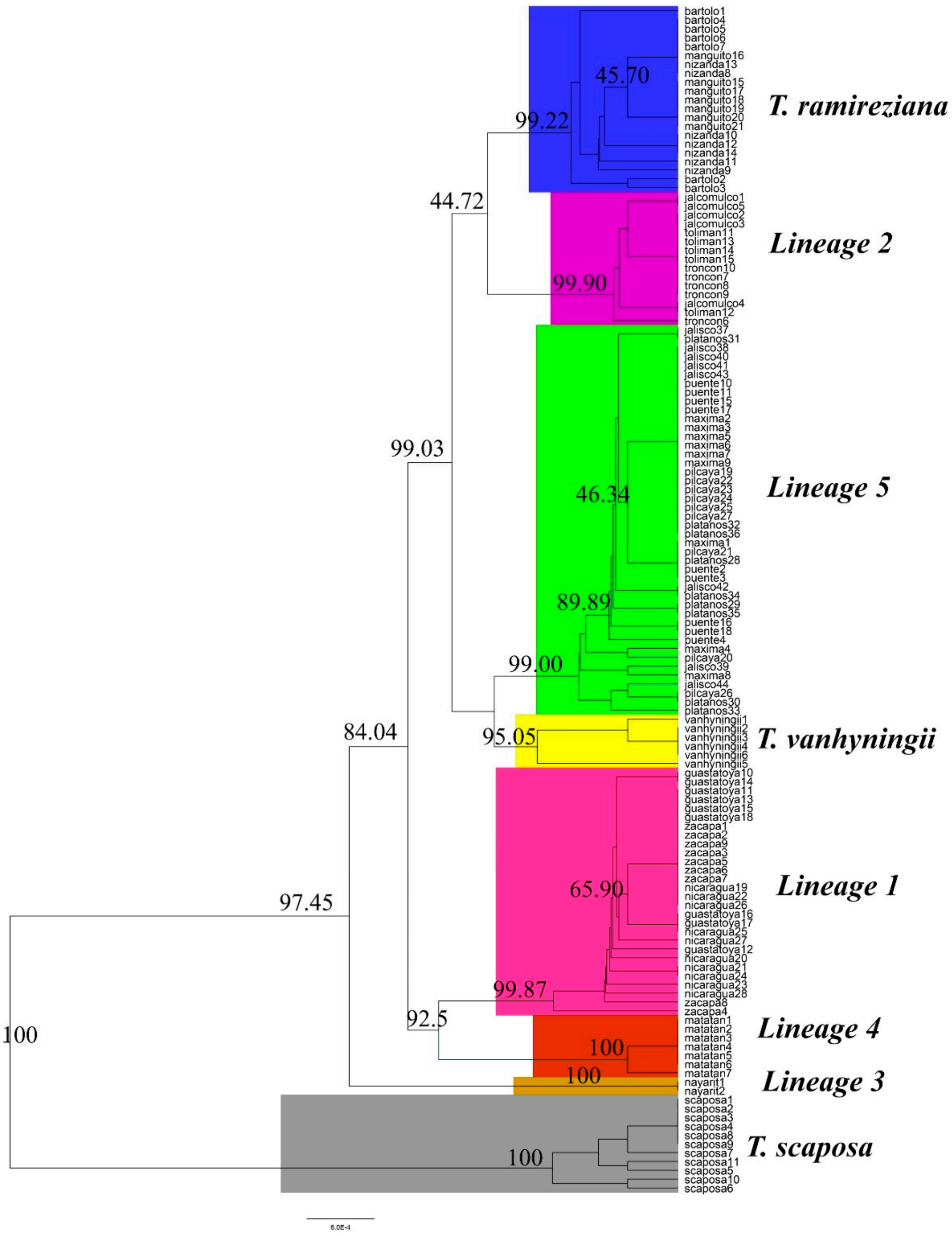

Figure S1. Gene tree derived from STACEY analysis for BEAST-2 using the *PHYC* marker sequences. PP values ≥0.90 on the branches.

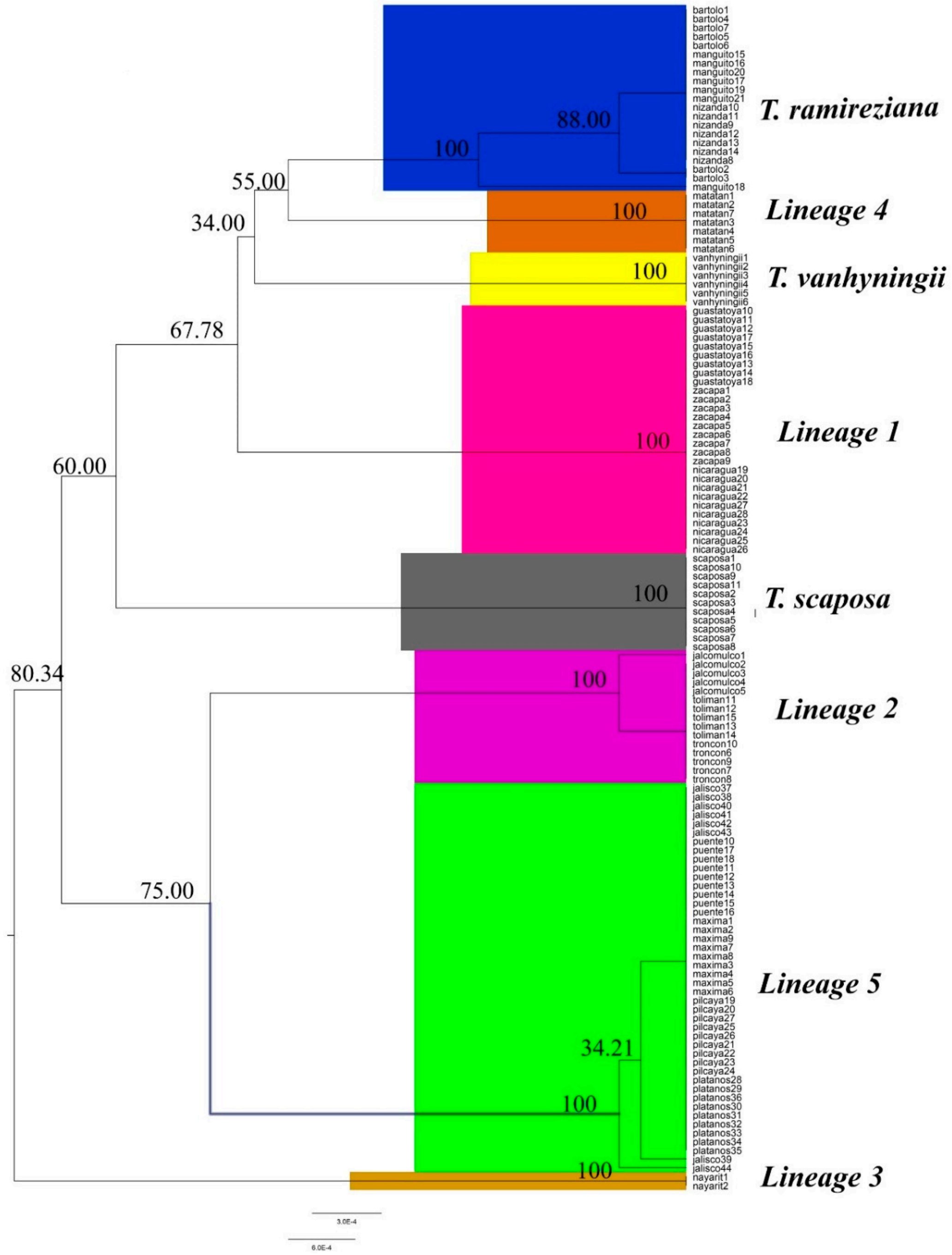

Figure S2. Gene tree derived from STACEY analysis for BEAST-2 using the *trnT-L-F* marker sequences. PP values ≥0.90 on the branches.

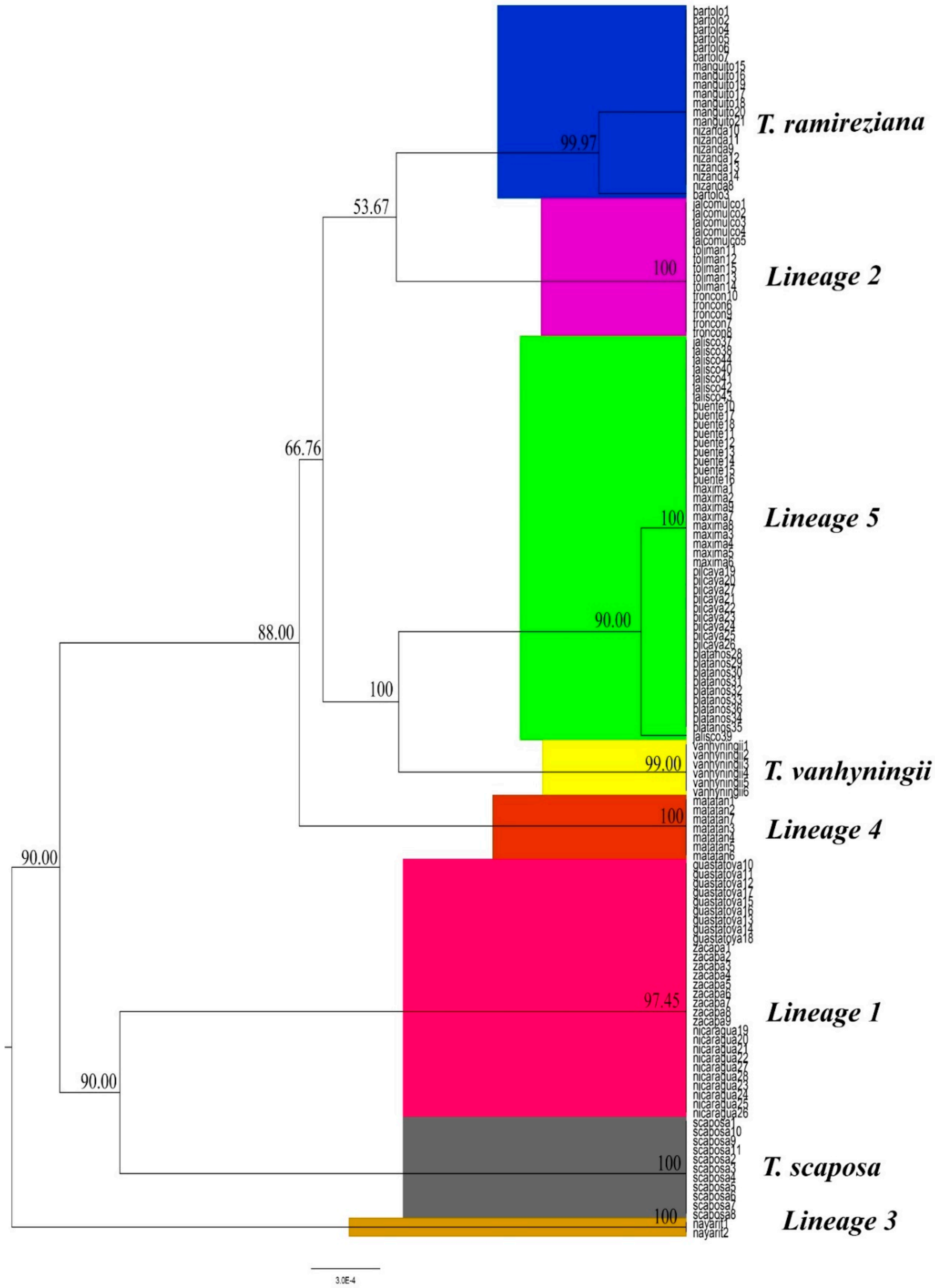

Figure S3. Gene tree derived from STACEY analysis for BEAST-2 using the *rps16-trnQ* marker sequences. PP values ≥0.90 on the branches.

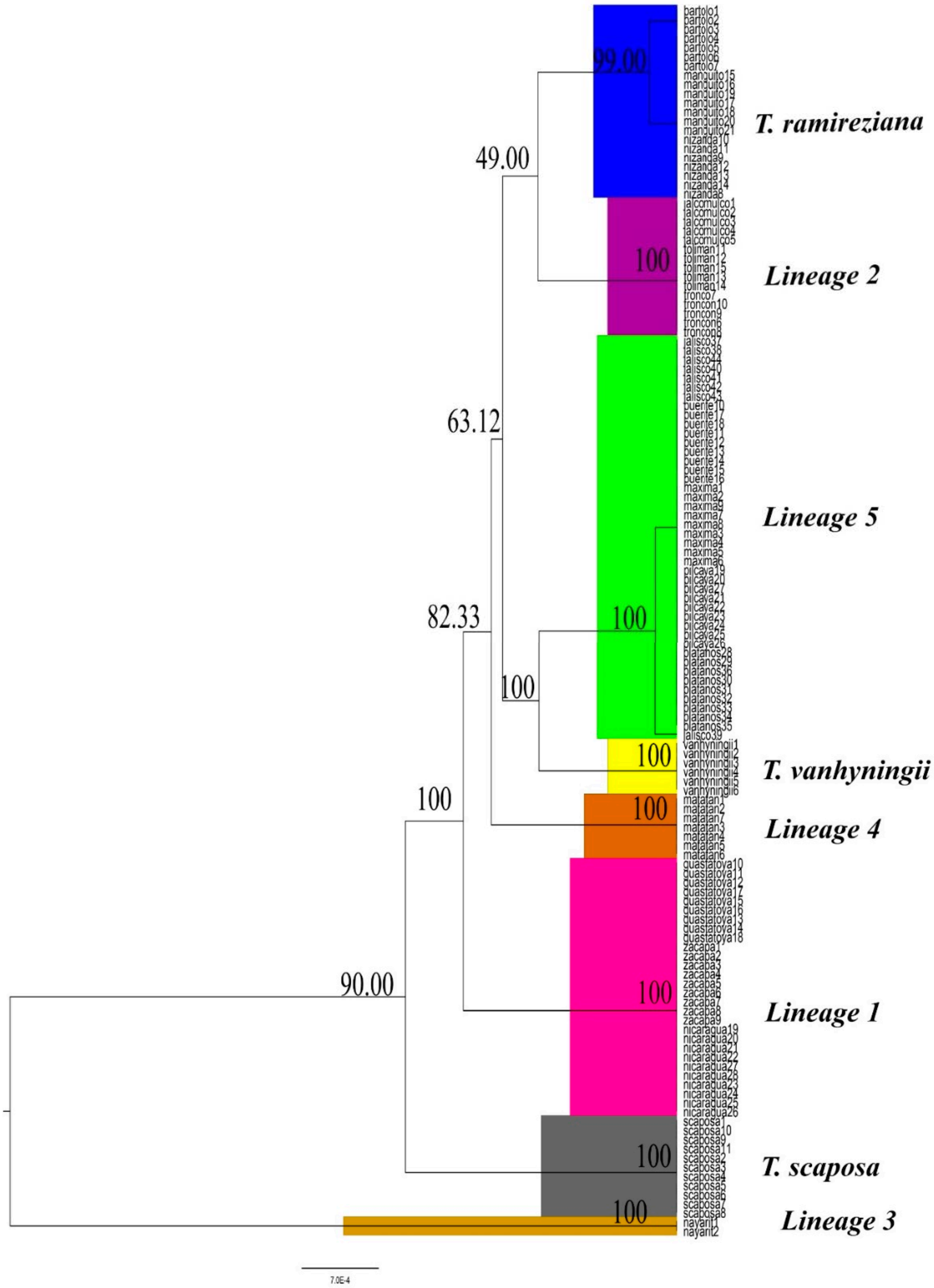

Figure S4. Gene tree derived from STACEY analysis for BEAST-2 using the *rpl16-rps3* marker sequences. PP values ≥0.90 on the branches.
